# Gram matrix: an efficient representation of molecular conformation and learning objective for molecular pretraining

**DOI:** 10.1093/bib/bbae340

**Published:** 2024-07-11

**Authors:** Wenkai Xiang, Feisheng Zhong, Lin Ni, Mingyue Zheng, Xutong Li, Qian Shi, Dingyan Wang

**Affiliations:** Lingang Laboratory, Shanghai 200031, China; Drug Discovery and Design Center, State Key Laboratory of Drug Research, Shanghai Institute of Materia Medica, Chinese Academy of Sciences, 555 Zuchongzhi Road, Shanghai 201203, China; University of Chinese Academy of Sciences, No. 19A Yuquan Road, Beijing 100049, China; Fujian Key Laboratory of Drug Target Discovery and Structural and Functional Research, School of Pharmacy, Fujian Medical University, Fuzhou 350122, China; Drug Discovery and Design Center, State Key Laboratory of Drug Research, Shanghai Institute of Materia Medica, Chinese Academy of Sciences, 555 Zuchongzhi Road, Shanghai 201203, China; Nanjing University of Chinese Medicine, 138 Xianlin Road, Nanjing 210023, China; Drug Discovery and Design Center, State Key Laboratory of Drug Research, Shanghai Institute of Materia Medica, Chinese Academy of Sciences, 555 Zuchongzhi Road, Shanghai 201203, China; University of Chinese Academy of Sciences, No. 19A Yuquan Road, Beijing 100049, China; Nanjing University of Chinese Medicine, 138 Xianlin Road, Nanjing 210023, China; Drug Discovery and Design Center, State Key Laboratory of Drug Research, Shanghai Institute of Materia Medica, Chinese Academy of Sciences, 555 Zuchongzhi Road, Shanghai 201203, China; University of Chinese Academy of Sciences, No. 19A Yuquan Road, Beijing 100049, China; Lingang Laboratory, Shanghai 200031, China; Lingang Laboratory, Shanghai 200031, China

**Keywords:** gram matrix, graph transformer, molecular property prediction

## Abstract

Accurate prediction of molecular properties is fundamental in drug discovery and development, providing crucial guidance for effective drug design. A critical factor in achieving accurate molecular property prediction lies in the appropriate representation of molecular structures. Presently, prevalent deep learning–based molecular representations rely on 2D structure information as the primary molecular representation, often overlooking essential three-dimensional (3D) conformational information due to the inherent limitations of 2D structures in conveying atomic spatial relationships. In this study, we propose employing the Gram matrix as a condensed representation of 3D molecular structures and for efficient pretraining objectives. Subsequently, we leverage this matrix to construct a novel molecular representation model, Pre-GTM, which inherently encapsulates 3D information. The model accurately predicts the 3D structure of a molecule by estimating the Gram matrix. Our findings demonstrate that Pre-GTM model outperforms the baseline Graphormer model and other pretrained models in the QM9 and MoleculeNet quantitative property prediction task. The integration of the Gram matrix as a condensed representation of 3D molecular structure, incorporated into the Pre-GTM model, opens up promising avenues for its potential application across various domains of molecular research, including drug design, materials science, and chemical engineering.

## Introduction

Small chemical molecules interact with biological macromolecules based on the principle of shape complementarity, forming the cornerstone of life processes regulation and drug therapy [1, 2]. Researchers are dedicated to studying molecule representations and properties [3–11], thereby advancing the drug discovery process [12]. These approaches, such as Attentive FP [13], D-MPNN [14], and TrimNet [6], primarily focus on using the graph convolutional neural network to represent molecular topological structure. Except for this, some strategies exist to improve model representational capability. Models relying on large-scale self-supervised pretraining like MG-BERT [5] may exhibit more robust performance under scenarios with limited labeled data. Additionally, models like NNPS [3], DSDP [4], and DRWBNCF [12], which utilize the biological profile of small molecules, provide valuable and associative information for downstream tasks such as drug repositioning and drug–drug interaction prediction. Each method has its unique strengths and weaknesses, tailored to different application contexts.

Among different modeling approaches, researchers have long recognized the significance of incorporating three-dimensional (3D) conformational information into molecular representation. Despite the existence of various methods for 3D characterization [15–18], they are often not the primary choice for molecular representation [19, 20], especially when compared to more widely known approaches such as molecular fingerprints [21–24] or 2D-topological graph [13, 25–35]. Challenges such as method complexity, compatibility issues with modeling approaches, and limited extraction of 3D information hinder the widespread adoption of 3D characterization. Consequently, there remains a need for the development of more efficient 3D characterization methods tailored for small molecules within the drug development community and among researchers.

The prevailing method for representing molecular topological information is through the use of molecular fingerprints [36]. Among these, the extended-connectivity finger-prints (ECFPs) [24] stand out as the most widely adopted, renowned for their ability to accurately depict underlying chemical substructures. To incorporate molecular 3D information, Seth *et al*. proposed a spherical extended 3D fingerprint (E3FP) [37] as an extension of the circular ECFP. E3FP not only retains the advantages of 2D topological fingerprints but also encodes 3D information in a faster way. However, since E3FP relies on principles of feature engineering, its performance may not consistently meet expectations for all tasks.

Subsequently, AI-based methods for extracting 3D conformational information from molecules have been developed. A notable example of these methods is the 3D equivariant neural network. Schnet [38] used successively filtered convolutional layers, enabling the model to obtain energy predictions that vary continuously with coordinates. DimeNet [39] introduced directional message passing, simultaneously considering the vector representation of the atom itself, interatomic distances, and bond angles, effectively leveraging directional information within the molecule. HMGNN [40] proposed a novel heterogeneous molecular graph representation that relied on interatomic distances and atomic numbers, featuring nodes and edges of different types to model many-body interactions. While 3D representations obtained through equivariant networks yield superior outcomes compared to molecular fingerprints, they suffer from several limitations. Principally, such representations are intricate and heavily reliant on precise conformational data, posing challenge in real-world scenarios where high-quality 3D data are often lacking. Fortunately, due to the success of pretrain and fine-tune pipelines [41, 42], some recent works have successfully encoded meaningful 3D information for downstream tasks through pretraining [43–45]. However, it is still challenging to convert this representation back and forth with the original 3D structure.

The 3D representation of proteins serves as a valuable reference for effective 3D information representation of molecules. Before the Alphafold era [46], protein structure prediction often relied on predicting a ‘Contact Map’ [47–49], an image representation that encodes the distance between each amino acid residue in a protein into a binary value. Despite being 2D, this representation provides effective conformational constraints for protein folding algorithms and has therefore been extensively utilized in protein structure prediction. Essentially, the Contact Map can be viewed as a compressed representation of the 3D coordinate information of a protein. Similarly, exploring the geometric space of individual compounds holds comparable significance. Adopting methods akin to protein structure prediction, the use of Distance Maps for molecules as a concise representation, satisfying the E(3)-group, emerges as a viable option. This approach offers simplicity compared to complex network-based encodings mentioned above, with the practical advantage of being convertible into coordinates through the Distance Map. While prior research has explored this concept [50, 51], the primary challenge lies in converting the Distance Map into coordinates, as this process requires the utilization of the Gram matrix [52].

Given that the Gram matrix serves as an intermediary variable for converting a Distance matrix into coordinates, this paper proposed using the Gram matrix directly as a molecular encoding for 3D conformation. Our approach offers several advantages compared to previous methods. Firstly, unlike methods employing equivariant networks for 3D representation, the Gram matrix is less complex and facilitates coordinate recovery via multidimensional scaling (MDS) [53], thus providing a more concise and systematic representation. Secondly, owing to the inherent properties of the Gram matrix, which is invariant to rotation and translation, similar to the Distance matrix, it exhibits greater robustness than coordinate-based representations and enhances compatibility with networks. Thirdly, our testing results indicate that the Gram matrix outperforms the Distance matrix as molecular representation in molecular prediction tasks, besides its capability to directly recover coordinates. Overall, the Gram matrix emerges as an excellent compressed representation of the 3D structure of molecules. In this study, considering the challenges in obtaining precise 3D structural information for molecules in real-world scenarios, we develop pretraining models to generate 3D structures via Gram matrix. This approach enables our model to derive 3D representations from stable 2D features, subsequently enhancing the predictive performance in downstream tasks.

Our work makes the following contributions:

(1) We propose for the first time that the Gram matrix can be utilized as a learning target for molecular pretraining, serving as a compressed representation of the 3D structure of molecules, and we demonstrate that the 3D structure of molecules can be swiftly reconstructed through the direct prediction of the Gram matrix.
(2) We observed that using the Gram matrix as the target for supervision during the pretraining phase resulted in superior outcomes compared to using the Distance matrix, bond length, and bond angle as the targets for supervision.
(3) We have developed a Graphormer-based model, referred to as pre-GTM, which utilizes the molecular representation from the Gram matrix-based pretraining stage. This model outperforms the benchmark Graphormer and other pretrained models in predicting quantitative properties on the Quantum Machines 9 (QM9) and MoleculeNet [54] datasets.

## Materials and methods

### Fundamentals of Gram matrix

The Gram matrix of 3D Cartesian coordinates serves as a compact and dense representation of molecular spatial information in this study. Given a conformer with $N$ atoms and corresponding origin-centered coordinate matrix $X\in{\mathbb{R}}^{N\times 3}$, the Gram matrix $G$ is defined as:


(1) \begin{equation*} {G}_{ij}={x}_i\cdot{x}_j \end{equation*}

where ${x}_i$ (${x}_j$) refers to the coordinate of the $i$-th ($j$-th) atom. For comparison, we introduce another similar approach to incorporating molecular 3D information: the Distance matrix $D$, defined as:


(2) \begin{equation*} {D}_{ij}=\left\Vert{x}_i-{x}_j\right\Vert =\sqrt{x_i^T{x}_i+{x}_j^T{x}_j-2{x}_i{x}_j} \end{equation*}

Combining Equations (1) and (2), $G$ and $D$ can be converted into each other:


(3) \begin{equation*} {D}_{ij}=\sqrt{G_{ii}+{G}_{jj}-2{G}_{ij}} \end{equation*}

(4) \begin{equation*} {G}_{ij}=\frac{1}{2}\left({D}_{0i}^2+{D}_{0j}^2-{D}_{ij}^2\right) \end{equation*}

where ${D}_{0i}$ (${D}_{0j}$) refers to the distance between the origin and the $i$-th ($j$-th) atom. It is interesting to observe from Equation (4) that ${G}_{ij}$ contains more information than ${D}_{ij}$, specifically ${D}_{0i}$ and ${D}_{0j}$. This can be considered one of the reasons why $G$ is a better representation than $D$.

### The conversion from Gram matrix to bond angles

Bond lengths and bond angles are two critical geometric parameters typically concerned during modeling [55, 56]. These parameters can also be directly derived from the Gram matrix. For the conversion to bond lengths, Equation (3) is sufficient, as bond length is a special case of atom distance. Equation (5) demonstrates how to convert $G$ into cosine values of any bond angles existing in the molecule:


(5) \begin{equation*} \cos{\angle}_{ij k}=\frac{G_{ik}+{G}_{jj}-{G}_{ij}-{G}_{jk}}{\sqrt{\left({G}_{ii}+{G}_{jj}-2{G}_{ij}\right)\left({G}_{jj}+{G}_{kk}-2{G}_{jk}\right)}} \end{equation*}

where ${\angle}_{ijk}$ denotes the degree of the bond angle connecting bonds ($i,j$) and ($j,k$).

**Figure 1:**
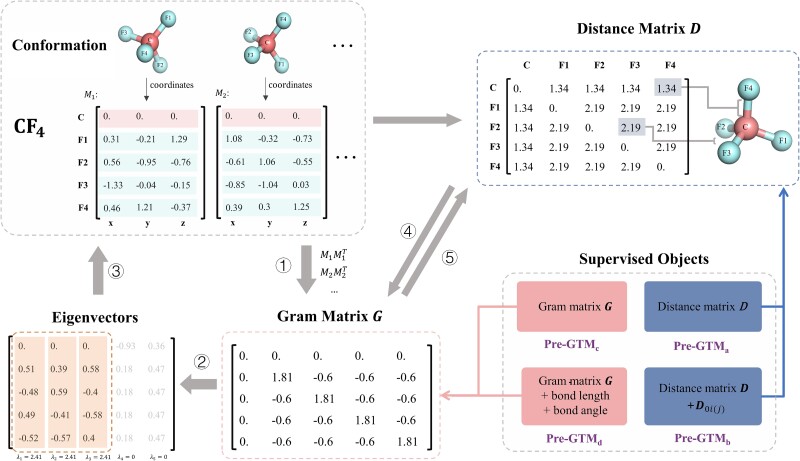
This graph provides a detailed demonstration of the MDS and the conversion between the Gram matrix $G$ and distance matrix $D$ using real values. The procedure involves the following steps: ① obtaining the Gram matrix from origin-centered coordinates results in a unique $G$, regardless of the rotation of the conformation. ② Decomposing $G$ into the eigenvectors and eigenvalues. ③ Restoring coordinates by selecting the three largest eigenvalues and their corresponding eigenvectors. ④ and ⑤ illustrate the interconversion between the $G$ and $D$. Besides, we demonstrate the four combinations of our supervised objects during pretraining.

Since $G$ is a compact representation of a 3D conformer, an important question arises: how do we reconstruct the corresponding conformation from a true or predicted $G$? A strategy named MDS can be employed to address this issue. A classical MDS method takes the Gram matrix as input and outputs the coordinates of the items that fulfill the constraint given by the input matrix; the procedure of MDS is illustrated in Fig. 1.

Specifically, for conformation reconstruction from $G$, eigen decomposition is utilized. As shown in Equation (6), $G$ is decomposed into the eigenvector matrix $Q$ and eigenvalue matrix $\Lambda$:


(6) \begin{equation*} G=Q\Lambda{Q}^{-1} \end{equation*}

For true $G$ that corresponds to a set of 3D coordinates, it can be proven that $G$ only has three positive eigenvalues ${\lambda}_1,{\lambda}_{2,}$ and ${\lambda}_3$ with others remaining to be zeros. Thus, the $k$-th coordinate of atom $i$ ($k=1,2\ or\ 3$) is given by:


(7) \begin{equation*} {x}_{ik}={\lambda}_k^{\frac{1}{2}}{Q}_{ik} \end{equation*}

Conversely, for predicted $G$ with inherent noise, the three largest eigenvalues and corresponding eigenvectors are selected to reconstruct the molecular conformation according to Equation (7).

## Model architecture

**Figure 2:**
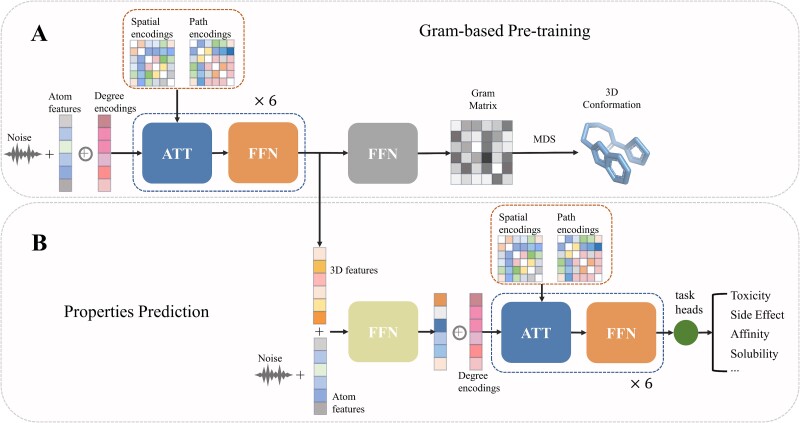
Summary of two stages in pre-GTM. (A) The pretraining stage. (B) The property prediction stage. Abbreviations: Pre-GTM, pretraining graph transformer model.

As demonstrated in Fig. 2, to illustrate the practical value of the Gram matrix, we propose a two-step procedure termed Pre-training Graph Transformers (Pre-GTM). Pre-GTM comprises the following steps:

(i) Pre-training Stage: This stage involves supervised pre-training with the Gram matrix (optionally together with bond lengths and angles) on an unlabeled training set with known geometry. Atom and bond features, as shown in Table 1, are explicitly incorporated into modeling on tasks without 3D information. (ii) Property Prediction Stage: In this stage, a molecular prediction model is trained on the labeled dataset, and predictions are made on the test set without known geometry. The 3D representations derived from the pretrained model are frozen and concatenated into the downstream model [57].

**Table 1:** Input features of our model.

Type	Feature	Size	Description
Atom	Atom number	1	Number of atoms
	Hybridization	5	Type of atom hybridization (sp, sp2, sp3, sp3d, sp3d2) (one-hot)
	Formal charge	1	Formal charge of atom
	Radical electrons	1	Number of radical electrons
	Van der Waals radius	1	Van der Waals radius
	Aromaticity	1	Whether this atom is part of an aromatic system
	Implicit hydrogens	1	Number of implicit hydrogens
	Explicit hydrogens	1	Number of explicit hydrogens
	Explicit valence	1	Explicit valence of atom
	Implicit valence	1	Implicit valence of atom
	Degree	1	Degree of the atom
	Chirality	2	The chirality type of an atom (one-hot)
Bond	Conjugated	1	whether or not the bond is considered to be conjugated
	Aromaticity	1	Whether this bond is part of an aromatic system
	Ring	1	Whether or not the bond is in a ring
	Bond type	4	Type of a bond (single, double, triple, aromatic) (one-hot)

### Pretraining stage

In the pretraining stage, a Graphormer [9] model ${\mathcal{M}}_a$ is employed to predict the Gram matrix on the pretraining dataset, as illustrated in Fig. 2A. To enhance the learning of representations related to 3D conformations, we also introduced bond length and bond angle prediction as auxiliary tasks. The input molecule is represented as an undirected graph $G=\left(\mathcal{V},\mathcal{E}\right)$ where the node set $\mathcal{V}$ corresponds to atoms and edge set $\mathcal{E}$ corresponds to chemical bonds, and then fed to the Graphormer model, a kind of graph neural network.

During the modeling with Graphormer, each atom $u\in \mathcal{V}$ is initialized with a state vector ${h}_u^0$, The central encoding ${z}_{\mathit{\deg}(u)}$ is computed based on $\deg (u)$ (the degree of atom u). The spatial encoding ${b}_{\phi \left(u,v\right)}$ is computed based on $\phi \left(\ u,v\right)$ (the shortest path between atom $u$ and $v$). Edge encoding ${c}_{ij}$ is calculated using the topology of the graph.

The provided encodings for pretraining in Graphormer involve several key steps. First, the atomic initial state vector ${h}_u^0$ and the central code ${z}_{\mathit{\deg}(u)}$ are added, and the resulting values are input into the Graphormer model to obtain the new atomic initial state vector ${h}_u^0$. Subsequently,${h}_u^0$ is normalized, and multihead attention is calculated to obtain the attention ${A}_{ij}$ between atomic pairs. This attention is then added to the spatial encoding ${b}_{\phi \left(u,v\right)}$ and the edge encoding ${c}_{ij}$, replacing the previous attention to form a new attention, also denoted as ${A}_{ij}$. The new attention ${A}_{ij}$ and${h}_u^0$ are residually connected, and the result is passed through a feedforward network block. This process is repeated until the specified number of network layers 𝑁 is reached, followed by a fully connected layer, ultimately yielding the final state vector ${h}_u$ of the atom. To directly predict ${G}_{uv}$ between any two nodes $u\in \mathcal{V}$ and $v\in \mathcal{V}$, we simply calculated the inner product between them:


(8) \begin{equation*} {\hat{G}}_{uv}={h}_u\bullet{h}_v \end{equation*}

where $\bullet$ denotes inner product. In order to improve the generalization of the model, we adopt a methodology from previous studies [58] by incorporating noise to differentiate between identical chemical environments. During the training phase, we add noise that obeys Gaussian distribution to the initial state vector ${h}_u^0$ for all atoms. During the testing phase, the same amount of noise added during the training phase can be applied.


(9) \begin{equation*} {h}_v^0\leftarrow{h}_v^0+{Z}_v\kern4.00em {Z}_v\sim \mathcal{N}\left(\mu, \sigma \right) \end{equation*}

where $\mu$ and $\sigma$ are the mean and variance of the Gaussian distribution, respectively.

The predicted $G$ is further used to predict the length of any bond ${l}_{uv}$and bond angle ${\Phi}_{uvw}$ where $\left(u,v\right)\in \mathcal{E}$ and $\left(v,w\right)\in \mathcal{E}$ using Equations (3) and (5), respectively. The unit of bond length ${l}_{uv}$ and bond angle ${\angle}_{uvw}$ is Å and rad, respectively. According to [43], the bond length and bond angle can also be predicted by the concatenation of ${h}_u$ and ${h}_v$:


(10) \begin{equation*} {l}_{uv}= MLP\left( CONCAT\left({h}_u,{h}_v\right)\right) \end{equation*}

(11) \begin{equation*} {\angle}_{uvw}= MLP\left( CONCAT\left({h}_u,{h}_v,{h}_w\right)\right) \end{equation*}

where $MLP$ refers to the multiple layer perceptron. The test results indicated that this method of constructing auxiliary tasks outperforms the method using Equations (3) and (5), as it is simpler and easy to converge.

In has been introduced that the Gram matrix can be directly transformed into the molecular coordinates through eigenvalue decomposition using Equation (6), which can be regarded as a conformation prediction model. Thus, we also calculated the root mean-squared deviation (RMSD) between the generated and true conformations in the test set to evaluate the performance of the minimum energy conformation prediction.

### Property prediction stage

In the property prediction stage, a new Graphormer model, denoted as ${\mathcal{M}}_b$, is built from scratch. It utilizes the atom embeddings acquired in the preceding stage to predict downstream molecular properties on a dataset without known geometry. This process is depicted in Fig. 2B.

To explicitly incorporate the geometric knowledge learned by ${\mathcal{M}}_a$ into the modeling process of ${\mathcal{M}}_b$, the final atom embedding ${h}_u$ for atom $u\in \mathcal{V}$, derived from the trained ${\mathcal{M}}_a$, remains fixed. It is then concatenated with the ${h}_u^{\prime }$ generated by ${\mathcal{M}}_b$, to compute the super node embedding ${h}_s$ of the molecule:


(12) \begin{equation*} {h}_s= READOUT\left({\left\{ CONCAT\Big( FROZEN\left({h}_u\right),{h}_u^{\prime}\Big)\right\}}_{u=1}^{\left|\mathcal{V}\right|}\right) \end{equation*}

Lastly, the super node embedding ${h}_s$ is passed into a fully connected layer to predict downstream tasks as a typical Graphormer does.

### Loss function

According to Equation (4), the Gram matrix ${G}_{ij}$ can be decomposed into two components: interatomic distances ${D}_{ij}$ and distances between atoms and the origin ${D}_{0i}$(${D}_{0j}$). Therefore, we propose three model settings:

① Directly supervising the Distance matrix (Pre-GTM_a_).

② Decomposing $G$ into the two components and supervising them separately (Pre-GTM_b_).

③ Directly supervising $G$ (Pre-GTM_c_).

The corresponding loss functions for the three models are shown in Equations (13)–(15), respectively. To incorporate global information, we use a super atom in Graphormer to represent the origin (0) in Equation (4). As a result, ${D}_{0i}$ can be obtained through a fully connected layer after concatenating the embedding vector of the super atom and the embedding vector of node $i$.


(13) \begin{equation*} Los{s}_a=\frac{1}{N^2}\sum_{j=1}^N\sum_{i=1}^N{\left({D}_{ij}-{\hat{D}}_{ij}\right)}^2 \end{equation*}

(14) \begin{equation*} Los{s}_b=\frac{1}{N^2}\sum_{j=1}^N\sum_{i=1}^N{\left({D}_{ij}-{\hat{D}}_{ij}\right)}^2+\frac{1}{N}\sum_{i=1}^N{\left({D}_{i0}-{\hat{D}}_{i0}\right)}^2 \end{equation*}

(15) \begin{equation*} Los{s}_c=\frac{1}{N^2}\sum_{j=1}^N\sum_{i=1}^N{\left({G}_{ij}-{\hat{G}}_{ij}\right)}^2 \end{equation*}

To explore whether the performance of the model can be further improved, we introduce two important geometric parameters, bond length and bond angle, on top of the supervised task in Pre-GTM_c_ and establish a new model, Pre-GTM_d_, with a corresponding loss function shown in Equation (16). Table 2 further details which supervision tasks correspond to each of the four models.

**Table 2:** Different combinations of supervised tasks.

Combinations	$G$	$D$	${d}_{0i}$	Bond length	Bond angle
Pre-GTM_a_		√			
Pre-GTM_b_		√	√		
Pre-GTM_c_	√				
Pre-GTM_d_	√			√	√

The $D$ and bond length are considered compatible as supervised targets. Abbreviations: $G$, Gram matrix; $D$, distance matrix.


(16) \begin{align*} Los{s}_d&=\frac{1}{N^2}\sum_{j=1}^N\sum_{i=1}^N{\left({G}_{ij}-{\hat{G}}_{ij}\right)}^2+\frac{1}{\left|\varepsilon \right|}\sum_{\left(i,j\right)\in \varepsilon }{\left({l}_{ij}-{\hat{l}}_{ij}\right)}^2 \nonumber \\&\quad +\frac{1}{\left|\mathcal{A}\right|}\sum_{\left(i,j,k\right)\in \mathcal{A}}\left({\angle}_{ij k}-{\hat{\angle}}_{ij k}\right) \end{align*}

where ${l}_{ij}$ denotes the length of the bond connecting atoms $i$ and $j$, with $\varepsilon$ as the set of bonds; ${\angle}_{ijk}$ denotes the degree of the bond angle connecting bonds ($i,j$) and ($j,k$), with $\mathcal{A}$ as set of angles.

### Baseline models

We primarily compare the performance of Pre-GTM with other baseline models in two application scenarios: molecular conformation generation and molecular property prediction. Different computational models are selected as baseline comparison models for each of these scenarios.

To assess molecular conformation prediction, the evaluation metric most relevant to drug design scenarios involves examining the similarity between predicted conformations and the ligand-binding conformations in protein–ligand cocrystal structures. Motivated by these considerations, we reference the work of Hou *et al*. [59] and compare the performance of our model with other conformation generation models based on the platinum diversity benchmark. The methods under comparison include traditional conformation prediction approaches (ConfGenX [60], Conformator [61], OMEGA [62], and RDKit) as well as six AI-based conformation prediction methods (ConfGF [63], DMCG [64], GeoDiff [65], GeoMol [58], torsional diffusion [66], and Uni-mol). Except for Uni-mol (a recently proposed universal 3D molecular representation learning framework), the metrics for other methods are directly taken from the original study by Hou *et al*.

In the context of molecular property prediction, we specifically compare Pre-GTM with other methods, particularly those that share similar application scenarios. These models are pretrained using molecular 3D conformation information and subsequently fine-tuned on downstream task datasets lacking conformation information through transfer learning. Among them, D-MPNN [34] and Graphormer [9] are widely used Graph neural network (GNN) architectures, and these models were trained from scratch. Hu *et al*. [67], N-Gram [68], and MolCLR [69] are pre-training methods, where molecular representations are generated in an unsupervised manner. DisPred predicts the distance between all atoms of the conformation with the highest probability (i.e. the lowest energy conformation). ConfGen is pretrained by generating up to 10 conformations. GraphCL is a traditional pretraining method based on data augmentation, requiring the model to learn to produce representations that are invariant to the augmentation of the data in a self-supervised manner. 3D Infomax [10] enforces the representation provided by a GNN model to incorporate latent 3D information by maximizing the mutual information between the GNN representation and 3D summary vectors. TransFoxMol incorporates a multiscale 2D molecular environment into a graph neural network + Transformer module and uses prior chemical maps to obtain a more focused attention landscape. Except for Graphormer and TransFoxMol, the metrics for other methods are directly taken from the original study of 3D Infomax.

### Metrics

We employed multiple evaluation metrics model comparison. These metrics encompassed the accuracy of predicting the Gram matrix, molecular conformation, and the quantitative properties across the QM9, GEOM-DRUGS, and MoleculeNet datasets.

We used R^2^, RMSE (root mean-squared error), and MAE (mean absolute error) to evaluate the performance of the model in predicting the Gram matrix. Additionally, the MAE metric was used to assess the model’s prediction of quantitative properties:


(17) \begin{equation*} {\mathrm{R}}^2=1-\frac{\sum{\left({y}_i-{\hat{y}}_i\right)}^2}{\sum{\left({y}_i-{\overline{y}}_i\right)}^2} \end{equation*}

(18) \begin{equation*} \mathrm{RMSE}=\sqrt{\frac{1}{N}{\sum}_{i=1}^N{\left({y}_i-{\hat{y}}_i\right)}^2} \end{equation*}

(19) \begin{equation*} \mathrm{MAE}=\frac{1}{N}{\sum}_{i=1}^N\left|{y}_i-{\hat{y}}_i\right| \end{equation*}

where ${y}_i$ represents the real value, ${\overline{y}}_i$ represents the mean value, and ${\hat{y}}_i$ represents the predicted value.

The RMSD, a standard measure of the difference between two molecular structures, was employed to evaluate the quality of the conformations generated by the model:


(20) \begin{equation*} \mathrm{RMSD}={\left(\frac{1}{N}{\sum}_{i=1}^n\left\Vert \phi \left({\hat{R}}_i\right)-{R}_i\right\Vert \right)}^{\frac{1}{2}} \end{equation*}

where $N$ represents the number of heavy atoms, $\phi$ is the function used for aligning two conformations by rotation and translation, and ${R}_i$ and ${\hat{R}}_i$ denote the coordinates of the true and the generated conformation, respectively.

The COV (coverage) and MAT (matching) metrics were utilized to quantify the quality of conformations [59]. The metrics are defined as:


(21) \begin{equation*} \mathrm{COV}\left({\mathbb{S}}_g\left(\mathcal{G}\right),{\mathbb{S}}_r\left(\mathcal{G}\right)\right)=\frac{\left|\left\{R\in{\mathbb{S}}_r|\mathrm{RMSD}\left(R,\hat{R}\right)<\delta, \exists \hat{R}\in{\mathbb{S}}_g\right\}\right|}{\left|{\mathbb{S}}_r\right|} \end{equation*}

(22) \begin{equation*} \mathrm{MAT}\left({\mathbb{S}}_g\left(\mathcal{G}\right),{\mathbb{S}}_r\left(\mathcal{G}\right)\right)=\frac{1}{\left|{\mathbb{S}}_r\right|}\sum_{R\in{\mathbb{S}}_r,\hat{R}\in{\mathbb{S}}_g}\min \mathrm{RMSD}\left(R,\hat{R}\right) \end{equation*}

where ${\mathbb{S}}_g$ and ${\mathbb{S}}_r$ are generated and reference molecular conformation ensembles for molecular $\mathcal{G}$, respectively. $\delta$ is a given RMSD threshold. COV assesses the diversity and detects the model-collapse phenomenon, while MAT measures the closeness between the generated and reference conformations. In our study, we limited the conformation ensembles size to 1 in Equations (21) and (22), as we only use them to assess the minimum energy conformation prediction.

### Complexity

In this section, we conducted an analysis of the algorithmic complexity of Pre-GTM. Suppose $N$ is the number of atoms, $k$ is the number of input features, $H$ is pre-GTM’s hidden size, and $L$ is the number of layers; the complexity of the embedding layer is $O\left(\left(N+k\right)H\right)$, the complexity of the attention layers is $O\left(L{H}^2\right)$, and the complexity of the feedforward layer is $O\left({N}^2H\right)$. Then, the complexity of pre-GTM is $O\left(L{H}^2+\left({N}^2+k\right)H\right)$.

## Materials

### Geometric Ensemble Of Molecules datasets

To thoroughly explore the representation capabilities of Gram matrix in various scenarios, our study employs two categories of molecules for pretraining, namely, small-sized molecules from the QM9 dataset [70] and drug-like molecules with a higher number of heavy atoms.

All molecular data are sourced from the Geometric Ensemble Of Molecules (GEOM) dataset [71]. This dataset comprises high-quality conformers for 133 258 molecules from the QM9 dataset. Additionally, it includes 304 466 drug-like species and their biological assay results, collectively known as GEOM-DRUGS dataset. These datasets were accessed as part of AICures (https://www.aicures.mit.edu). Table 3 provides summary statistics of the molecules constituting the dataset. The drug-like molecules from AICures are typically medium-sized organic compounds, with an average of 44.4 atoms (24.9 heavy atoms) and a maximum of 181 atoms (91 heavy atoms). These molecules exhibit significant variability, as evidenced by the mean (6.5) and maximum (53) number of rotatable bonds. In contrast, the QM9 dataset is constrained to 9 heavy atoms (C, O, N, and F) and 29 total atoms, with a much smaller molecular mass and few rotatable bonds.

**Table 3:** Dataset details: number of (heavy) atoms and rotatable bonds.

GEOM-DRUGS dataset (*N* = 304 466)			
	Mean	Standard deviation	Maximum
Number of atoms	44.4	11.3	181
Number of heavy atoms	24.9	5.7	91
Number of rotatable bonds	6.5	3	53
**QM9 dataset (*N* = 133 258)**			
	**Mean**	**Standard deviation**	**Maximum**
Number of atoms	18	3	29
Number of heavy atoms	8.8	0.51	9
Number of rotatable bonds	2.2	1.6	8

QM9 was utilized to compare the property prediction performance of different molecular representation methods. Quantum mechanical properties and spatial information (the lowest energy conformation) were computed using the density-functional theory (DFT) method. Quantitative properties and spatial information were directly obtained from the MoleculeNet.

To ensure the fairness in model evaluation, the entire QM9 and GEOM-DRUGS dataset was partitioned into training, validation, and test sets as a ratio of 8:1:1. Moreover, to prevent data leakage in the downstream task, we removed duplicate molecules from the GEOM-DRUGS dataset that were identical to those in the downstream tasks.

### MoleculeNet datasets

In this study, two molecular regression datasets and seven molecular classification datasets from the MoleculeNet dataset were chosen as benchmark datasets. Details regarding these datasets are provided in Table 4. They cover various fields including physical chemistry, physiology, and biophysics, as outlined in Table 4. ESOL [72] is a standard dataset containing water solubility for common organic small molecules, It is extensively employed in the development of deep learning–based models for predicting water solubility. The Lipo (Lipophilicity) dataset was sourced from the ChEMBL database, which includes experimental results for the octanol or water partition coefficient (logP), a commonly used measure of a molecule’s solubility. The human immunodeficiency virus (HIV) database includes over 40 000 molecules that have been experimentally assessed for their ability to inhibit HIV replication. The BACE database provides predicted results for the activity of human β-protease inhibitors. The Binary labels of Blood-brain Barrier Penetration (BBBP) [73] dataset contains information on the permeability of the blood–brain barrier. The TOX21 dataset comprises toxicity testing data for compounds against 12 distinct targets, including nuclear receptors and cell signaling pathways. ToxCast serves as a repository of toxicological data for thousands of molecules, providing numerous toxicity annotations for a wide range of chemicals through high-throughput screening experiments. Additionally, Side Effect Resource (SIDER) is a collection of marketed drugs and adverse drug reactions (ADRs). ClinTox [74] is a database of U.S. Food and Drug Administration (FDA) approved drugs and drugs that failed clinical trials due to toxicity. For property prediction tasks on these datasets, we adhere to the recommended scaffold splitting methods, which have been shown to be more practically useful [54].

**Table 4:** Dataset details: number of compounds and tasks, splits, and metrics.

Category	Dataset	Task type	Tasks	Compounds	Split	Metric
Physical chemistry	ESOL	Regression	1	1128	Scaffold	RMSE
	Lipophilicity	Regression	1	4200	Scaffold	RMSE
Biophysics	HIV	Classification	1	41 913	Scaffold	AUC-ROC
	BACE	Classification	1	1522	Scaffold	AUC-ROC
Physiology	BBBP	Classification	1	2053	Scaffold	AUC-ROC
	Tox21	Classification	12	8014	Scaffold	AUC-ROC
	ToxCast	Classification	617	8615	Scaffold	AUC-ROC
	SIDER	Classification	27	1427	Scaffold	AUC-ROC
	ClinTox	Classification	2	1491	Scaffold	AUC-ROC

## Results

### *G* is E(3)-invariant representation of three-dimensional coordinates

In this section, we discuss the invariant properties of Gram matrix and the outcomes of converting the true Gram matrix to atom coordinates.

Given the geometric nature of 3D molecule, it is often desirable for a method encoding spatial information to be equivariant or invariant with respect to E(3)-group, encompassing rotation, translation, and reflection (inversion and mirroring). E(3)-invariant implies that the spatial encoding remains unchanged under these transformations or any finite combination thereof [75]. Clearly, the Gram matrix is E(3)-invariant. According to Equation (1), the Gram matrix represents the inner product of origin-centered coordinates, which inherently remains constant under the aforementioned transformations. This renders the Gram matrix an optimal means of encoding molecular 3D coordinates.

Subsequently, we utilize all samples from QM9 to verify that the molecular conformation can effectively be reconstructed from the true Gram matrix through eigen decomposition. We calculate the RMSD between the conformations generated by each molecule using the actual $G$ and their corresponding actual conformations, yielding an average value of $1.603\times{10}^{-8} $Å.

**Figure 3:**
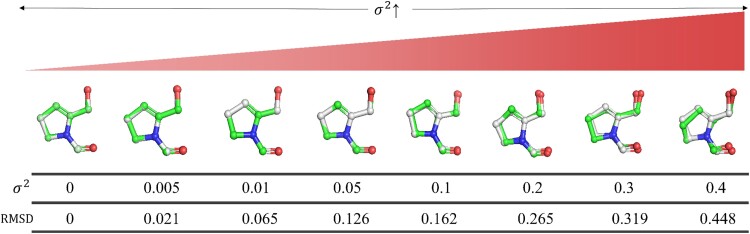
To a certain degree, noise can be tolerated when reconstructing coordinates via MDS. Conformations obtained by $G$ or $G$ with added noise via MDS are aligned with true conformations. The $\sigma$ is the deviation of Gaussian noise. Abbreviations: MDS, multidimensional scaling; $G$, Gram matrix.

MDS demonstrates proficiency in reconstructing coordinates from an accurate Gram matrix and exhibits a degree of resilience to noise within the Gram matrix. To affirm this, we introduce noise to the Gram matrix with varying variances but consistent means, as depicted in Fig. 3. As observed in the first row of Fig. 3, MDS is capable of accommodating a Gram matrix with a certain degree of noise when the variance of the noise is relatively small. Theoretically, after the eigen decomposition, MDS only considers the first three largest eigenvalues and their corresponding eigenvectors according to Equation (7). For a true Gram matrix, all eigenvalues except the first three largest ones are zero. However, with a noisy Gram matrix, multiple nonzero eigenvalues may emerge, potentially altering the order and size of the first three largest eigenvalues, thereby leading to inaccuracies in the resulting coordinates.

### Learning $G$ is a good proxy task for three-dimensional conformation generation

After demonstrating the accuracy of coordinate transformation using the precise Gram matrix, in this section, we further discuss the predictability of the Gram matrix on the QM9 and GEOM-DRUGS dataset.

To enhance the model’s ability to predict molecular conformations, we have incorporated a variety of auxiliary tasks. Consequently, we assessed different combinations of these tasks and developed four distinct models, as outlined in Table 2. The precise combinations of auxiliary tasks allocated to each model are thoroughly elucidated in the Materials and Methods section.

Table 5 presents the performance of these four models in predicting $G$. Additionally, we include RDKit [76, 77] as a baseline for comparison. This study draws four notable conclusions:

**Table 5:** The results of predicting$G$, $D$, bond length, and bond angle using different combinations of supervised tasks on the QM9 and GEOM-DRUGS dataset.

QM9						
Method	Length	Angle	$ \boldsymbol D$	$\boldsymbol G$		Molecule
	MAE	MAE	MAE	*R* ^2^	MAE	RMSD(Å)
RDKit						0.586
Pre-GTM_a_	0.069 ± 0.005	0.158 ± 0.012	0.120 ± 0.018	0.645 ± 0.023	1.145 ± 0.026	0.645 ± 0.029
Pre-GTM_b_	0.089 ± 0.004	0.143 ± 0.007	0.107 ± 0.014	0.814 ± 0.009	0.704 ± 0.015	0.485 ± 0.012
Pre-GTM_c_	0.104 ± 0.009	0.115 ± 0.005	0.083 ± 0.002	0.961 ± 0.009	0.344 ± 0.025	0.337 ± 0.010
Pre-GTM_d_	**0.065 ± 0.003**	**0.083 ± 0.002**	**0.078 ± 0.003**	**0.970 ± 0.005**	**0.246 ± 0.017**	**0.241 ± 0.007**
**GEOM-DRUGS**						
**Method**	**Length**	**Angle**	$ \boldsymbol D$	$\boldsymbol G$		**Molecule**
	**MAE**	**MAE**	**MAE**	***R* ^2^**	**MAE**	**RMSD(Å)**
RDKit						2.251
Pre-GTM_a_	0.392 ± 0.018	0.484 ± 0.016	1.327 ± 0.036	0.446 ± 0.051	5.711 ± 0.157	3.011 ± 0.038
Pre-GTM_b_	0.417 ± 0.020	0.477 ± 0.018	0.948 ± 0.033	0.636 ± 0.024	4.271 ± 0.046	2.247 ± 0.052
Pre-GTM_c_	0.474 ± 0.026	0.706 ± 0.041	0.877 ± 0.047	0.740 ± 0.006	3.862 ± 0.014	1.722 ± 0.039
Pre-GTM_d_	**0.243 ± 0.014**	**0.431 ± 0.011**	**0.747 ± 0.004**	**0.761 ± 0.011**	**3.690 ± 0.013**	**1.619 ± 0.018**

The best performance is marked in bold.

Abbreviations: $G$, Gram matrix; $D$, distance matrix; MAE, mean absolute error; RMSD, root mean-squared deviation.

Directly supervising the Gram matrix $G$ through Pre-GTM_c_ yields precise predictions of the Gram matrix of molecular coordinates, with an *R*^2^ value of 0.961 on the QM9 dataset and 0.74 on the GEOM-DRUGS dataset. This indicates that the graph neural network can accurately predict the Gram matrix of molecular coordinates.

Compared with supervising Distance matrix (Pre-GTM_a_) or separately supervises ${D}_{ij}$, ${D}_{0i}, and\ {D}_{0j}$ (Pre-GTM_b_), the model directly supervised with the Gram matrix (Pre-GTM_c_) performs better. This aligns with intuition, as the direct prediction of the Gram matrix entails less computational complexity than initially predicting the Distance matrix and subsequently utilizing Equations (3) and (4) to derive the Gram matrix.

Pre-GTM_d_, integrating auxiliary tasks such as bond length and bond angle into Pre-GTM_c,_ demonstrates a substantial enhancement in the precision of predicting $G$. The MAE associated with the prediction outcomes decreased from 0.344 to 0.242 on the QM9 dataset and from 1.722 to 1.619 on the GEOM-DRUGS dataset. This underscores the significance of bond length and bond angle, crucial geometric parameters, in facilitating conformation prediction.

Models Pre-GTM_c_ and Pre-GTM_d_ demonstrated significant improvement compared to the RDKit baseline, while models Pre-GTM_a_ and Pre-GTM_b_ do not exhibit advantages.

Aside from the Gram matrix $G$, Table 5 presents metrics for the Distance matrix, bond lengths, bond angles, and molecular conformations. While the four Pre-GTM models have different prediction targets, these targets ($G$, $D$, bond length, and bond angle) can be interconverted. Consequently, we assessed the prediction error for all targets across each model setting. To more clearly represent model performance, we also provided the RMSD of the predicted molecular structures for each model. It is evident that Pre-GTM_d_ demonstrates superior performance across all prediction tasks. In conclusion, appropriate auxiliary tasks (bond lengths and bond angles) and learning objectives (Gram matrix) are crucial elements in ensuring the predictive accuracy of the model.

To compare the effectiveness of conformation prediction models, we evaluated the performance of our Pre-GTM_d_ model alongside other conformation prediction methods using a test dataset of 3354 high-quality ligand bioactive conformations [59]. And the results are summarized in Table 6. Here we set the Maximum Ensemble Size to 1 for comparison as our model only utilized the lowest energy conformation for training. As we can see, Pre-GTM_d_ performed best on the COV metric compared to other AI models, but does not perform as well as the traditional methods and performs close to Conformator. In terms of the MAT metric, Pre-GTM_d_ slightly underperformed GeoMol and torsional diffusion, mainly due to higher prediction errors for larger molecules.

**Table 6:** Qualities of generated conformer in terms of mean COV (%). RMSD threshold δ = 2.00 Å. Maximum ensemble size is set to 1.

Method category	Method	COV(%)	MAT(Å)
AI methods	ConfGF [63]	39.42	2.377
	DMCG [64]	46.06	2.2
	GeoDiff [65]	57.13	1.91
	Torsional diffusion [66]	63.95	1.742
	GeoMol [58]	65.98	1.695
	Uni-mol [7]	68.85	**1.651**
	Pre-GTM_d_	**69.29**	1.769
Traditional computational methods	Conformator	69.65	1.549
	RDKit	70.45	1.599
	ConfGenX	73.43	1.51
	OMEGA	73.46	1.48

Abbreviations: RMSD, root mean-squared deviation; COV, coverage; MAT, matching.

Our method is marked in bold. The best performance is marked in bold.

**Figure 4:**
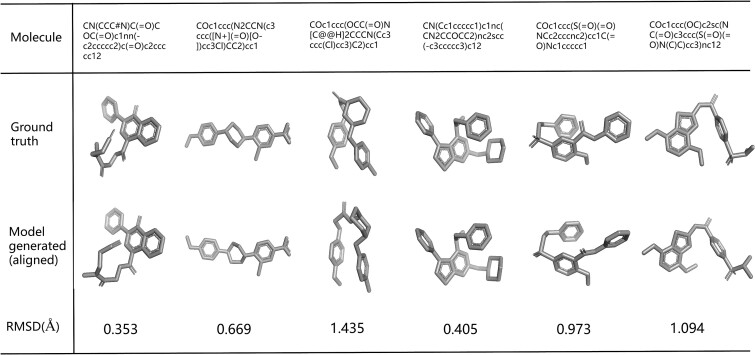
Conformation prediction instances from GEOM-DRUGS dataset demonstrate that the conformations returned by the model (Pre-GTM_d_) closely align with the true conformations.

We provide six instances of employing Pre-GTM_d_ for predicting the minimum energy conformation of molecules in GEOM-DRUGS dataset (Fig. 4). It is apparent that the ground truth and the model generated conformation exhibit close alignment.

**Figure 5:**
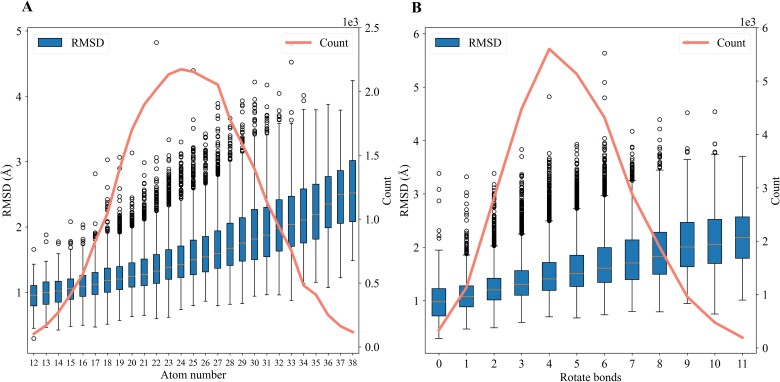
(A) The correlation between atom number and RMSD. (B) The association between the number of rotatable bonds in a molecule and RMSD. Box-and-whisker plots show the median (center line), 25th, and 75th percentile (lower and upper boundary), with 1.5× inter-quartile range indicated by whiskers and outliers shown as individual data points. Groups with fewer than 100 samples were excluded from the analysis. Abbreviations: RMSD, root mean-squared deviation.

In addition to assessing Pre-GTM’s predictive capability on the Gram matrix within the QM9 and GEOM-DRUGS datasets, we investigated the impact of atom number and the number of rotatable bonds in a molecule on the accuracy of conformational prediction. This was achieved by evaluating RMSD between the conformation reconstructed from the predicted Gram matrix and the true conformation of the molecule. Figure 5 illustrates the impact of atom number (Fig. 5A) and rotatable bonds (Fig. 5B) on RMSD. The red line represents the distribution of the number of atom number (Fig. 5A) and rotatable bonds (Fig. 5B) on the test split of the GEOM-DRUGS dataset (groups with fewer than 100 samples are not displayed), while the blue box illustrates their effect on RMSD. As demonstrated in Fig. 5A, with the increase in atom count from 12 to 38, the model’s capacity to predict molecular conformation gradually diminishes, indicating that larger molecules present greater difficulty for prediction. Additionally, Fig. 5B illustrates that with the increase in the number of rotatable bonds from 0 to 11, the model’s predictive capability for molecular conformation gradually diminishes. This suggests that molecules with greater flexibility pose a greater challenge for prediction.

## Graph neural network pretrained with $G$ improves molecular representation learning

The significance of pretraining via Gram matrix prediction was further underscored by evaluating the performance of Pre-GTM in downstream tasks, encompassing eight tasks within the QM9 dataset and property prediction tasks across nine datasets in MoleculeNet.

### Quantum Machines 9 downstream tasks

We evaluated Pre-GTM’s performance in predicting the eight tasks of QM9. The comparative outcomes are summarized in Table 7. The Pre-GTM model showed superior performance compared to models like Graphormer and D-MPNN trained from scratch, providing strong evidence for its effectiveness. Additionally, Pre-GTM outperformed self-supervised learning models such as N-Gram, highlighting the importance of using Gram matrix for pretraining and leveraging 3D geometric information in subsequent tasks. Moreover, Pre-GTM_d_ outperformed Pre-GTM_c_, indicating that enhanced 3Drepresentation leads to better property prediction.

**Table 7:** Results of properties prediction on the QM9 dataset (MAE). Pre-GTM_c_ denotes the utilization of the Gram matrix loss during pretraining, while Pre-GTM_d_ signifies the utilization of the Gram matrix, bond length, and bond angle loss during pretraining.

task	mu	alpha	HOMO	LUMO	gap	R2	ZPVE	Cv
Unit	D	Bohr^3^	meV	meV	meV	Bohr^2^	meV	cal/mol K
D-MPNN [34]	0.45 ± 0.01	0.493 ± 0.01	93 ± 5.00	106 ± 2.00	148 ± 3.00	24.37 ± 0.92	37 ± 4.00	0.24 ± 0.01
N-Gram [68]	0.54 ± 0.00	0.611 ± 0.02	142 ± 1.00	138 ± 1.00	193 ± 1.00	59.14 ± 0.18	9 ± 0.00	0.33 ± 0.01
Hu *et al*. [67]	0.54 ± 0.00	1.725 ± 0.01	116 ± 0.00	118 ± 0.00	161 ± 1.00	55.42 ± 0.29	83 ± 1.00	0.71 ± 0.01
MolCLR [69]	0.46 ± 0.00	0.463 ± 0.02	87 ± 0.00	92 ± 0.00	127 ± 0.00	17.43 ± 0.92	33 ± 4.00	0.16 ± 0.00
3D infomax [10]	0.35 ± 0.01	0.327 ± 0.01	69 ± 0.32	**70 ± 0.54** ^*^	102 ± 2.03	17.39 ± 0.94	**8 ± 1.87** ^*^	0.13 ± 0.01
Pre-GTM_c_	0.35 ± 0.01	0.250 ± 0.02	67 ± 2.39	75 ± 4.84	96 ± 5.03	17.63 + 1.33	11 ± 0.73	**0.12 ± 0.01**
Pre-GTM_d_	**0.33 ± 0.00** ^*^	**0.244 ± 0.01**	**66 ± 1.26**	74 ± 2.21	**95 ± 1.37**	**16.38 ± 0.52** ^*^	13 ± 1.67	**0.12 ± 0.01** ^*^

The best performance is marked in bold.

All models were run five times with different random seeds. Two-sided *t*-test was applied between the models, and the exact *P*-values are in source data. (Note: ^*^P ≤ .01)

Abbreviations: MAE, mean absolute error.

### MoleculeNet downstream tasks

We investigated the performance of the Pre-GTM_d_ model on property prediction tasks using the MoleculeNet dataset, which consists of drug-like molecules. The results of the comparison between Pre-GTM_d_ and the benchmark models are presented in Table 8. Pre-GTM_d_ significantly outperforms all other models, including the randomized control, on approximately half of downstream tasks. This finding suggests that the 3D characterization learned through the use of the Gram matrix is beneficial for predicting the properties of drug-like molecules.

**Table 8:** Results of properties prediction on the MoleculeNet dataset.

Metric	Dataset	DisPred [9]	ConfGen [9]	GraphCL [11]	3D Infomax [10]	TransFoxMol [8]	Rand Init	Pre-GTM_d_
RMSE	ESOL	0.986 ± 0.03	0.867 ± 0.05	0.959 ± 0.05	0.894 ± 0.03	0.972 ± 0.05	0.896 ± 0.04	**0.859 ± 0.03**
	Lipo	0.718 ± 0.02	0.757 ± 0.04	0.714 ± 0.01	0.695 ± 0.01	**0.689 ± 0.02**	0.808 ± 0.01	0.738 ± 0.01
AUC-ROC	BBBP	66.06 ± 1.84	66.16 ± 2.24	**71.06 ± 2.00** ^*^	69.1 ± 1.07	68.25 ± 0.74	64.33 ± 1.79	68.47 ± 1.61
	Tox21	73.87 ± 0.43	75.24 ± 1.00	**78.92 ± 0.61** ^*^	74.46 ± 0.74	73.21 ± 0.79	71.39 ± 0.62	72.52 ± 0.55
	ToxCast	61.58 ± 0.58	64.74 ± 1.20	64.95 ± 0.31	64.41 ± 0.88	62.81 ± 2.3	68.13 ± 0.52	**69.31 ± 0.69** ^*^
	SIDER	57.13 ± 1.89	56.34 ± 4.20	57.32 ± 5.00	53.37 ± 3.34	57.2 ± 1.3	54.16 ± 2.59	**59.54 ± 1.83** ^*^
	ClinTox	55.77 ± 5.86	64.27 ± 5.22	51.07 ± 5.52	59.43 ± 3.21	**85.8 ± 1.54** ^*^	61.83 ± 2.77	78.68 ± 3.26
	HIV	75.66 ± 1.26	76.57 ± 1.39	76.06 ± 1.06	76.08 ± 1.33	66.45 ± 1.31	71.27 ± 1.40	**76.74 ± 0.84**
	BACE	76.51 ± 1.95	80.02 ± 1.58	77.18 ± 4.01	79.42 ± 1.94	**83.74 ± 1.83** ^*^	71.54 ± 1.25	82.04 ± 1.48

The best performance is marked in bold.

All models were run five times with different random seeds. Two-sided *t*-test was applied between the models, and the exact *P*-values are in source data. (Note: ^*^P ≤ .01)

## Conclusion

In this study, we introduce an innovative integration of Graphormer with the Gram matrix, enabling the generation of 3D molecular representations entirely from 2D structure. Furthermore, by utilizing the learned precise 3D representations on the QM9 and MoleculeNet datasets, we enhance the performance of quantitative property prediction tasks.

Despite the success demonstrated by Pre-GTM, there are still various opportunities for improvement across multiple areas, including but not limited to pretraining with more extensive datasets. Our current pretraining approach relies on the QM9 and GEOM-DRUGS datasets (which provide 3D coordinates), limited by their small data size, thus hindering the model’s generalization ability for predicting 3D representations. To overcome this limitation, it is crucial to explore datasets with increased samples and larger molecular sizes, such as PCQM4Mv2 [78]. Furthermore, the complexity is growing at a quadratic level of the number of heavy atoms $N$ and hidden size $H$, affecting model’s efficiency and performance. This issue could be addressed by implementing local predictions or switch to a framework simpler than Graphormer. Lastly, there is potential to adapt the model architecture and information integration for the prediction of molecular conformational distributions or the generation of conformations. These enhancements could further advance the model’s capabilities in molecular property prediction tasks.

**Key Points**

- We propose a graph transformer model, termed Pre-GTM, to predict the 3D structure and properties of drug-like molecules.
- Supervising Gram Matrix (E(3)-invariant) is a good way to acquire high-quality 3D representations.
- The learned 3D representations in pretraining stage enhance the molecular property prediction.
- We illustrate the advanced performance of Pre-GTM on drug-like datasets compared to other supervised methods for 3D structure and properties inference.

## Data and code availability

The GEOM dataset is available at https://dataverse.harvard.edu/dataset.xhtml?persistentId=doi:10.7910/DVN/JNGTDF. The 3354 high-quality ligand bioactive conformations from Hou *et al*. are available at https://github.com/wangzhehyd/fastsmcg/tree/main. The MoleculeNet dataset is available at https://moleculenet.org/. The codes used to build the Pre-GTM are available in the GitHub repository (https://github.com/xiangwenkai/GRAM).

## Author Biographies

**Wenkai Xiang** is working as a researcher in Lingang Laboratory. His current interests include graph neural network, natural language processing, and bioinformatics.

**Feisheng Zhong** obtained his PhD Degree at Shanghai Institute of Materia Medica. His research interest is artificial intelligence–assisted molecular representation.

**Lin Ni** obtained her Master Degree at Nanjing University of Chinese Medicine and Shanghai Institute of Materia Medica. Her research interest is molecular representation learning.

**Mingyue Zheng** is a professor at Shanghai Institute of Materia Medica. His research interest is the development of accurate drug design techniques based on artificial intelligence, computational chemistry, and computational biology.

**Xutong Li** is an associate professor at Shanghai Institute of Materia Medica. Her primary research interest is the development of accurate drug design techniques based on artificial intelligence, including multi-target selective drug design, drug–target interactions, and explainable deep learning for druggability prediction.

**Qian Shi** is a researcher at Lingang Laboratory. Her research interest is artificial intelligence–assisted drug design.

**Dingyan Wang** is a researcher at Lingang Laboratory. His research interest is artificial intelligence–assisted drug design.
